# Global Pattern of CD8^+^ T-Cell Infiltration and Exhaustion in Colorectal Cancer Predicts Cancer Immunotherapy Response

**DOI:** 10.3389/fphar.2021.715721

**Published:** 2021-09-10

**Authors:** Sun Tian, Fulong Wang, Rongxin Zhang, Gong Chen

**Affiliations:** ^1^ Carbon Logic Biotech (HK) Limited, Hongkong, China; ^2^ StateKey Laboratory of Oncology in South China, Department of Colorectal Surgery, Sun Yat-sen University Cancer Center, Collaborative Innovation Center for Cancer Medicine, Guangzhou, China

**Keywords:** biomarker, prediction method, colorectal cancer, cancer immunotherapy response, anti-PD1

## Abstract

**Background:** The MSI/MSS status does not fully explain cancer immunotherapy response in colorectal cancer. Thus, we developed a colorectal cancer-specific method that predicts cancer immunotherapy response.

**Methods:** We used gene expression data of 454 samples (MSI = 131, MSI-L = 23, MSS = 284, and Unknown = 16) and developed a TMEPRE method that models signatures of CD8+ T-cell infiltration and CD8^+^ T-cell exhaustion states in the tumor microenvironment of colorectal cancer. TMEPRE model was validated on three RNAseq datasets of melanoma patients who received pembrolizumab or nivolumab and one RNAseq dataset of purified CD8^+^ T cells in different exhaustion states.

**Results:** TMEPRE showed predictive power in three datasets of anti-PD1-treated patients (p = 0.056, 0.115, 0.003). CD8^+^ T-cell exhaustion component of TMEPRE model correlates with anti-PD1 responding progenitor exhausted CD8^+^ T cells in both tumor and viral infection (p = 0.048, 0.001). The global pattern of TMEPRE on 454 colorectal cancer samples indicated that 10.6% of MSS patients and 67.2% of MSI patients show biological characteristics that can potentially benefit from anti-PD1 treatment. Within MSI nonresponders, approximately 50% showed insufficient tumor-infiltrating CD8^+^ T cells and 50% showed terminal exhaustion of CD8^+^ T cells. These terminally exhausted CD8^+^ T cells coexisted with signatures of myeloid-derived suppressor cells in colorectal cancer.

**Conclusion:** TMEPRE is a colorectal cancer-specific method. It captures characteristics of CD8^+^ T-cell infiltration and CD8^+^ T-cell exhaustion state and predicts cancer immunotherapy response. A subset of MSS patients could potentially benefit from anti-PD1 treatment. Anti-PD1 resistance MSI patients with insufficient infiltration of CD8^+^ T cells or terminal exhaustion of CD8^+^ T cells need different treatment strategies.

## Introduction

Immune checkpoint inhibitors produce durable responses in some microsatellite instable (MSI) colorectal cancer patients. However, still, approximately 60% of MSI colorectal cancer patients do not respond to single immune checkpoint inhibitor treatment such as anti-PD1, and approximately 40% of MSI colorectal patients do not respond to combinations of immune checkpoint inhibitor treatment (Oliveira et al., 2019). The mechanism of resistance is unclear. In colorectal cancer, MSI/MSS status is widely used as an indication of whether a patient should receive immunotherapy. Therefore, most studies on colorectal cancer were focused on the comparison between MSI tumors and MSS tumors. Although these studies provide insights into the difference between these two colorectal cancer subtypes, they do not explain why resistance to immune checkpoint inhibitor treatment occurs within MSI colorectal tumors. In addition to MSI/MSS status, other biomarkers such as TMB, PDL1, POLE/POLD1 mutation, or MSI-like gene signature are also used in colorectal cancer (Tian et al., 2012; Havel et al., 2019). Essentially, PDL1 provides a direct indication of whether a tumor sample of a colorectal cancer patient has high CD8^+^ T-cell infiltration, while MSI/MSS status, TMB, POLE/POLD1 mutation, and MSI-like gene signature characterize the likelihood of a tumor sample generating high neoantigen level, thus indirectly indicating whether a tumor sample of a colorectal cancer patient could potentially have high CD8^+^ T-cell infiltration. However, it is already evident from the studies of anti-PD1 response in lung cancer and melanoma that the number of tumor-infiltrating CD8^+^ T cells is not the only requirement of response to anti-PD1 treatment; the characteristics of exhaustion state of tumor-infiltrating CD8^+^ T cells is also required (Thommen et al., 2015; Li et al., 2019; Sade-Feldman et al., 2019). Therefore, regardless of how technically robust biomarkers such as MSI/MSS status, TMB, PDL1, POLE/POLD1 mutation, and MSI-like gene signature, these biomarkers will only characterize the quantity of tumor-infiltrating CD8^+^ T cells. However, the quantity of tumor-infiltrating CD8^+^ T cells alone will not fully explain anti-PD1 resistance in colorectal cancer. There is a lack of prediction method of anti-PD1 response in colorectal cancer.

It is known that a tumor at least has two well documented immune escape mechanisms to become resistant to anti-PD1 treatment: lack of CD8^+^ T-cell infiltration and CD8^+^ T-cell dysfunction (Sharma et al., 2017; Sade-Feldman et al., 2019; Yost et al., 2019; Wu et al., 2020). Therefore, a colorectal tumor should meet at least two characteristics to become a responder to anti-PD1 treatment an anti-PD1 treatment responding tumors should have CD8^+^ T-cell infiltration and at least a subset of tumor-infiltrating CD8^+^ T cells display properties that can respond to anti-PD1. In this report, we develop a TMEPRE method that dissects the gene expression patterns of these two characteristics from the tumor microenvironment in colorectal cancer and predict anti-PD1 response.

## Materials and Methods

### Data Used for the Development of the TMEPRE Model

Publicly available gene expression data with known MSI/MSS status of four colorectal cancer datasets [GSE13294 (Jorissen et al., 2008), GSE26682 (Vilar et al., 2011), GSE18088 (Gröne et al., 2011), and GSE39084 (Kirzin et al., 2014)] were downloaded from GEO database. All four datasets are from the same Affymetrix Human Genome U133 Plus 2.0 Array platform, and normalization was performed using the frozen RMA (fRMA) method in the *frma* package (McCall et al., 2010). The batch effects of samples in four datasets were removed using *ComBat* (Johnson et al., 2007). In total, gene expression data of 454 samples were collected (MSI = 131, MSI-L = 23, MSS = 284, Unknown = 16). The public or the patients were not involved in the design, conduct, reporting, or dissemination plans of our research.

### Design of the TMEPRE Model

The score function of the TMEPRE model is comprised of two components: TME1.TcellInfiltration and TME2.TcellResponse.

1) TME1.TcellInfiltration scores tumor microenvironment that allows CD8^+^ T-cell infiltration. To estimate the abundance of CD8^+^ T cells, we use the expression level of CD8A. The cutoff of CD8A level is defined as 40% percentile of CD8A expression level in 131 MSI tumors. MSI tumors with a CD8A level higher than the cutoff are classified as tumors with high CD8^+^ T-cell infiltration (n = 78); MSS tumors with a CD8A level lower than the cutoff are classified as tumors without high CD8^+^ T-cell infiltration (*n* = 211). 200 rounds of 10-fold cross-validation between these two groups were performed. In each cross-validation round, a *t*-test for each gene was performed and p-values of genes were ranked. CD8A gene itself was excluded from the cross-validation procedure. Genes with p-values in the top 60 ranked genes in at least 80% of 200 rounds of cross-validations were selected as the signature of TME1.TcellInfiltration *.* The selected genes were used to construct the nearest centroid method. The inputs are expression values of these selected genes and the output is a TME1.TcellInfiltration score. To ensure that all potential anti-PD1 responders are selected sensitively, the cutoff was optimized to maximize the sensitivity and minimize the false-negative rate.

The relative range coverage ω of TME1.TcellInfiltration scores of MSS samples is defined as follows:

$$\omega =\left(\max.score_{mss}-\min.score_{mss}\right)/\left(\max.score_{all}-\min.score_{all}\right).$$

2) TME2.TcellResponse scores tumor microenvironment, that tumor-infiltrating CD8^+^ T cells do not display a terminal exhaustion pattern and can still respond to checkpoint inhibitors. To define the terminal exhaustion pattern of tumor-infiltrating CD8^+^ T cells, we use the co-expression pattern of multiple inhibitory receptors of PD1 and TIM3 because TIM3 is an early acquired co-expressed inhibitor receptor among all co-expressed inhibitory receptors and using an early co-inhibitory receptor, TIM3, could sensitively capture more tumor samples with terminal exhausted CD8^+^ T cells (Thommen et al., 2015). Within MSI tumors with high CD8^+^ T-cell infiltration defined in the previous step (*n* = 78), the median of PD1 expression level is used as the cutoff of PD1 and the median of TIM3 expression level is used as the cutoff of TIM3. MSI tumors with high CD8^+^ T-cell infiltration and both PD1 and TIM3 higher than cutoffs are defined as a tumor microenvironment of co-expression of multiple early inhibitory receptors. CD8^+^ T cells in this type of tumor microenvironment become terminally exhausted and resist anti-PD1 treatment (*n* = 21). MSI tumors with high CD8^+^ T-cell infiltration but both PD1 and TIM3 lower than cutoffs are defined as a tumor microenvironment in which CD8^+^ T cells can still respond to anti-PD1 treatment (*n* = 21). 200 rounds of 10-fold cross-validation between these two groups were performed. In each cross-validation round, a *t*-test for each gene was performed and p-values of genes were ranked. Genes with p-values in the top 60 ranked genes in at least 80% of 200 rounds of cross-validations were selected as the signature of TME2.TcellResponse *.* The selected genes were used to construct the nearest centroid method. The inputs are expression values of these selected genes and outputs are a TME2.TcellResponse score. To ensure that all potential anti-PD1 responders are selected sensitively, the cutoff was optimized to maximize the sensitivity and minimize the false-negative rate.

### Data Used for Testing Predictive Values of the Anti-PD1 Response of the TMEPRE Model

To test the predictive value of the anti-PD1 response, three RNAseq datasets were used. The first dataset includes normalized RNAseq data and clinical data of pretreatment samples of melanoma patients who received pembrolizumab or nivolumab. Patients in this cohort who received MAPK inhibitor were removed (*n* = 16, GSE78220) (Hugo et al., 2016). The second dataset includes normalized RNAseq data and clinical data of samples of melanoma patients who received nivolumab. Samples at the early treatment time point before cycle 1 day 29 and samples at the pretreatment time point before cycle 1 day 0 were analyzed separately. Patients who received *a priori* ipilimumab treatment or with incomplete overall survival data were removed (n = 21, GSE91061) (Riaz et al., 2017). The platform of these three anti-PD1-treated patients’ datasets is different from the platform of the datasets used in the development of TMEPRE and the cutoff values can not be directly used. In three datasets of anti-PD1-treated patients, the median was therefore used as the default cutoff. TMEPRE comprises two components; the median was equally split as the cutoffs for each component. A colorectal tumor with either a low TME1.TcellInfiltration score (lowest 25%) or a high TME1.TcellInfiltration score (highest 25%) but a low TME2.TcellResponse score is considered as an anti-PD1 nonresponder. Survival analysis was performed using the R package survival (Therneau and Grambsch, 2000). The hazard ratio was calculated using the Cox proportional-hazards regression model and the *p*-value was calculated using the log-rank test. The combined *p*-value of three validation sets was calculated using the *Z*-transform method implemented in the R package survcomp (Whitlock, 2005; Haibe-Kains et al., 2008).

To read the TME2.TcellResponse score of the TMEPRE model on exhausted CD8^+^ T cells**,** a dataset including normalized RNAseq data from progenitor exhausted and terminally exhausted CD8^+^ T cells isolated from tumors and chronic viral infection (n = 20, GSE122713) was used (Miller et al., 2019). Because the TME2.TcellResponse signature is derived from the gene expression data of bulk tumor sample, the source of gene expressions originates from a mixture of CD8^+^ T cells, tumor cells, and other tumor-infiltrating immune cell types in the tumor microenvironment, while the progenitor/terminal exhausted tumor-infiltrating CD8^+^ T cells data are generated from isolated CD8^+^ T cells. When TME2.TcellResponse scores were read, only genes in TME2.TcellResponse that primarily originated from CD8^+^ T cells are used. For each gene in TME2.TcellResponse, median expression values in 16 purified main immune cell types were compared using BloodSpot with HemaExporer human hematopoiesis database (Bagger et al., 2019). A gene is considered as mainly expressed by CD8^+^ T cells when CD8^+^ T cell is among the top two immune cell types expressing this gene. The score function used for the read-out is the nearest centroid.

## Results

### TMEPRE Model Predicts the Anti-PD1 Treatment Response

The TMEPRE model was developed using gene expression data of colorectal cancer patients and has two components: TME1.TcellInfiltration (28 genes, Supplementary Table S1) and TME2.TcellResponse (29 genes, Supplementary Table 2).

**FIGURE 1:**
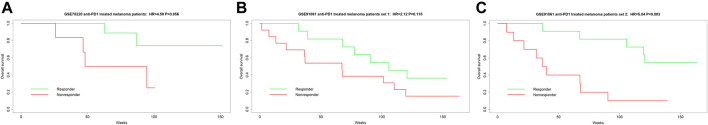
TMEPRE was validated on three datasets of melanoma patients who received anti-PD1 treatment. **(1A)** n = 16, pretreatment samples, patients were treated with pembrolizumab or nivolumab, HR = 4.59, *p*-value = 0.056; **(1B)** n = 21, sampling before cycle 1 day 0, patients were treated with nivolumab, HR = 2.12, *p*-value = 0.115; **(1C)** n = 21, sampling at an early treatment time point before cycle 1 day 29, patients were treated with nivolumab, HR = 5.04, *p*-value = 0.003. Separation of overall survival between TMEPRE-predicted responder group and TMEPRE-predicted nonresponder group is clear in all three validations.

To date, melanoma is widely used as the main prototypic cancer type of immune hot tumor to study cancer immunotherapy response. Thus, most of the available gene expression datasets of anti-PD1 response were performed using melanoma as the model system. Because TMEPRE is a method that mainly measures characteristics of the tumor microenvironment and different cancer types have shared tumor microenvironment characteristics, in our study, we also used anti-PD1-treated melanoma datasets as a model system to test predictive values of TMEPRE. TMEPRE was validated on three datasets of melanoma patients who received anti-PD1 treatment. In the first dataset, the survival analysis of the TMEPRE prediction model resulted in a significant hazard ratio (*n* = 16, pretreatment samples, GSE78220, HR = 4.59, *p*-value = 0.056, Figure 1A). In the second dataset, although the *p*-value of the survival analysis of the TMEPRE prediction model is large (n = 21, sampling before cycle 1 day 0, GSE91061, HR = 2.12, *p*-value = 0.115, Figure 1B), the separation of survival between the TMEPRE-predicted responder group and the TMEPRE-predicted nonresponder group is still clearly observed. In the third dataset, the survival analysis of the TMEPRE prediction model resulted in a significant hazard ratio (n = 21, sampling at an early treatment time point before cycle 1 day 29, GSE91061, HR = 5.04, *p*-value = 0.003, Figure 1C). The sample sizes of anti-PD1-treated samples in the current publicly available databases are small, and this small sample size artificially increased the *p*-values of each individual log-rank test (Ioannidis, 2019; Thiese et al., 2016). Still, the trend of separation of the responder group and the nonresponder group is clear in all three validation sets (Figures 1A–C). When three validation sets are combined together, the *Z*-transform combined probability test showed a significant *p*-value (*p*-value = 0.0007) (Whitlock, 2005). Taken together, the survival analysis indicated clinical significance, and the TMEPRE model showed predictive values for anti-PD1 treatment response.

### The Underlying Biology of the TMEPRE Model Measures Amounts of Tumor-Infiltrating CD8^+^ T Cells and Characteristics of Tumor-Infiltrating Terminally Exhausted CD8^+^ T Cells

**FIGURE 2:**
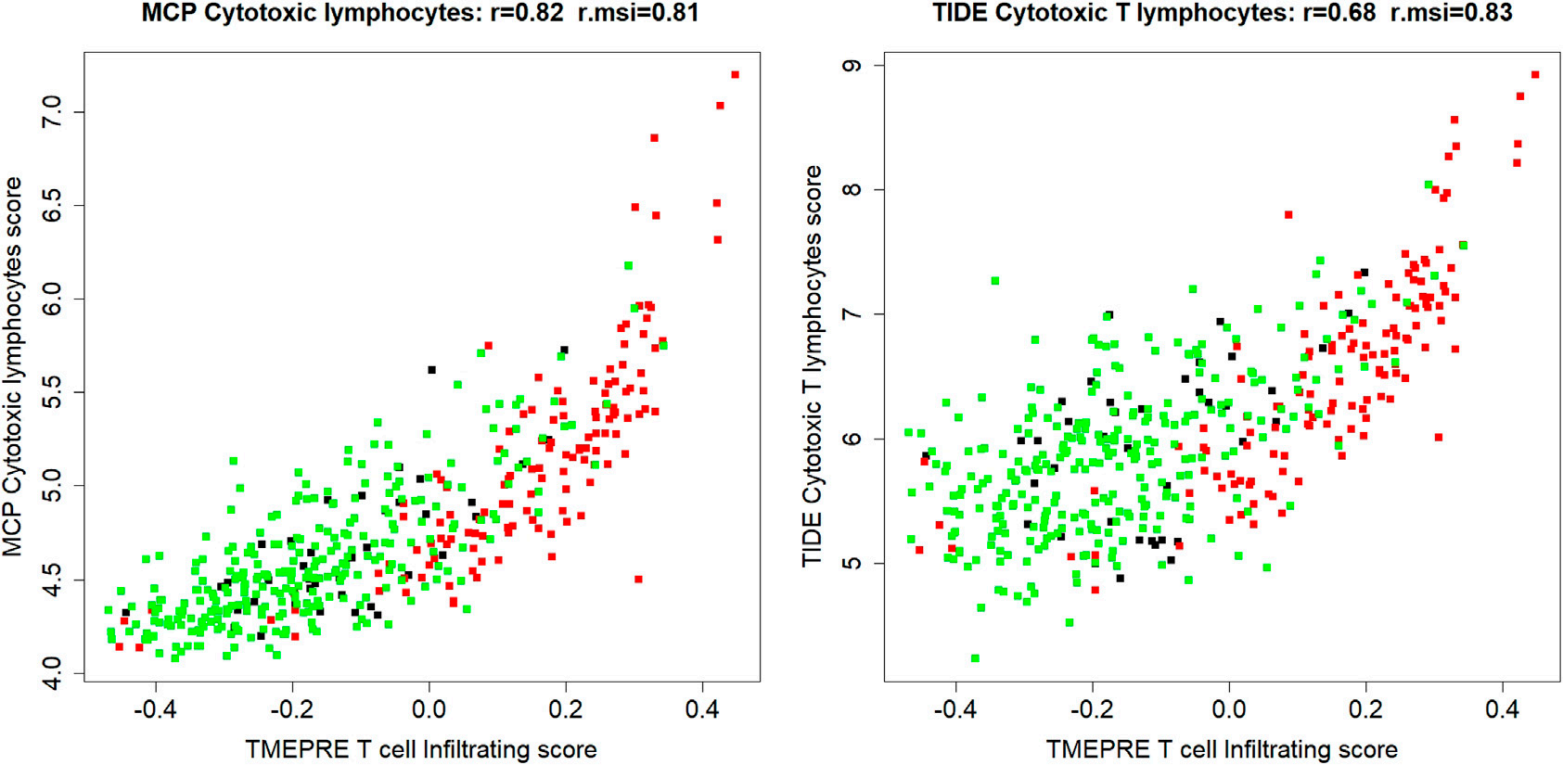
In 454 colorectal cancer samples (MSI = 131, MSI-L = 23, MSS = 284, Unknown = 16), the first component of the TMEPRE model**,** TME1.TcellInfiltration score (*x*-axis), positively correlates with the counting of MCP-counter cytotoxic lymphocytes score (*y*-axis, left panel) and TIDE cytotoxic T lymphocytes score (*y*-axis, right panel). MSI tumors are shown in red; MSS tumors are shown in green; MSI-L and MSI-unknown tumors are shown in black. The relative range coverage of TME1.TcellInfiltration scores of MSS samples ($\omega_{MSS.TME1.TcellInfiltration}$=0.89) is the largest among all three methods.

In the dataset of all 454 samples, the counts of tumor-infiltrating cytotoxic lymphocytes were read out using MCP-counter and TIDE cytotoxic T lymphocytes count (Becht et al., 2016; Jiang et al., 2018). The first component of the TMEPRE model**,** TME1.TcellInfiltration score, positively correlates with counting of MCP-counter cytotoxic lymphocytes (*r* = 0.82, r. msi = 0.81) and TIDE cytotoxic T lymphocytes (r = 0.68, r. msi = 0.83) (Figure 2). The relative range coverage of the TME1.TcellInfiltration scores of MSS samples is larger than the relative range coverage of MCP-counter score and TIDE score ($\omega_{MSS.TME1.TcellInfiltration}$=0.89, $\omega_{MSS.MCP.\text{Cytotoxiclymphocyte}}\;$ = 0.67, $\omega_{MSS.TIDE.\text{CytotoxicTlymphocyte}}\;$ = 0.81). These results suggested that counting of cytotoxic T lymphocytes by TME1.TcellInfiltration tends to agree with other counting methods, but in MSS colorectal tumors that harbor fewer tumor-infiltrating immune cells, TME1.TcellInfiltration might be a more sensitive measurement of tumor-infiltrating cytotoxic lymphocytes. The reason might be that TME1.TcellInfiltration is specifically designed for the tumor microenvironment of colorectal cancer.

**FIGURE 3:**
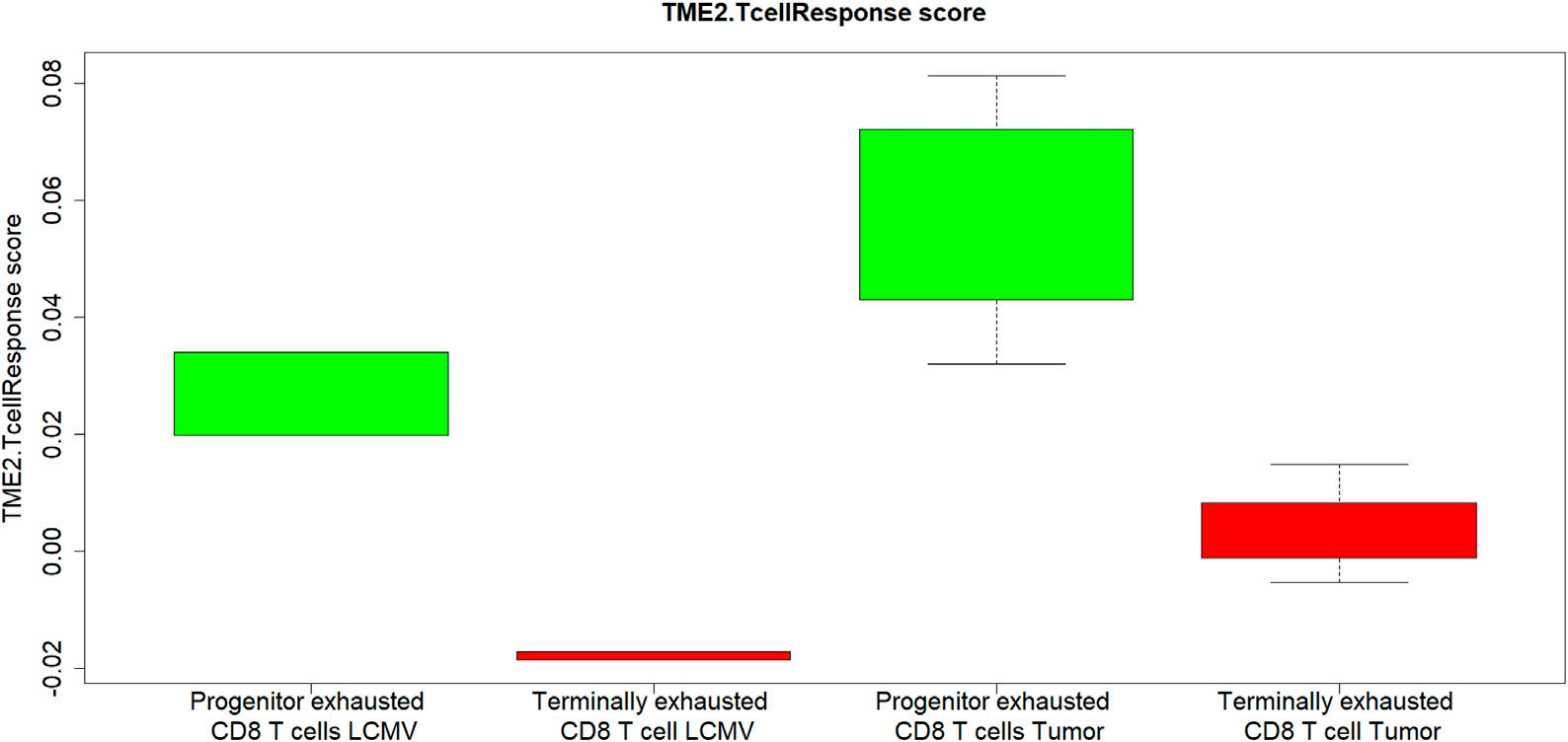
In both tumor and chronic viral infection, CD8^+^ T cells components of TME2.TcellResponse significantly correlates with anti-PD1 responding progenitor exhausted CD8^+^ T cells (*p* = 0.048, 0.001, respectively). The *X*-axis is the groups of CD8^+^ T cells. Green is the purified progenitor exhausted CD8^+^ T cells; red is the purified terminally exhausted CD8^+^ T cells. *Y*-axis is the TME2.TcellResponse score calculated using CD8^+^ T cells components of TME2.TcellResponse.

The second component of the TMEPRE model**,** TME2.TcellResponse score, is designed to measure whether tumor-infiltrating CD8^+^ T cells can respond to anti-PD1 treatment. To test whether TME2.TcellResponse indeed captures this characteristic of tumor-infiltrating CD8^+^ T cells, we read out the scores of TME2.TcellResponse signature in two subgroups of dysfunction CD8^+^ T cells isolated from tumors and chronic viral infection: terminally exhausted tumor-infiltrating CD8^+^ T cells that can no longer respond to anti-PD-1 therapy and progenitor exhausted tumor-infiltrating CD8^+^ T cells that can still respond to anti-PD-1 therapy (GSE122713) (Miller et al., 2019; Busselaar et al., 2020). Only genes in TME2.TcellResponse that likely primarily originated from CD8^+^ T cells are used to read out scores on gene expression data generated from isolated CD8^+^ cells. Seven genes (CCL5, CD2, CD48, CD84, FAM78A, HCST, and IL21R) in TME2.TcellResponse are considered as mainly expressed by CD8^+^ T cells as CD8^+^ T cell is among the top two immune cell types expressing them in the BloodSpot HemaExporer human hematopoiesis database (Bagger et al., 2019). Two genes in TME2.TcellResponse are inhibitor receptors on CD8^+^ T cells used to define early terminal exhausted CD8^+^ T cells (HAVCR2, PDCD1). These nine genes were used to read out TME2.TcellResponse scores in the isolated progenitor/terminal exhausted tumor-infiltrating CD8^+^ T cells dataset. In both tumors and chronic viral infection, the scores of TME2.TcellResponse are significantly higher in the subgroup of progenitor exhausted tumor-infiltrating CD8^+^ T cells ($pvalue_{tumor}$ <0.001, $pvalue_{viralinfection}$= 0.048, Figure 3). Therefore, the score of TME2.TcellResponse indeed captures the characteristics of progenitor exhausted tumor-infiltrating CD8^+^ T cells that can still respond to anti-PD1.

### Global Pattern of the TMEPRE Model in MSI and MSS Colorectal Tumors

**FIGURE 4:**
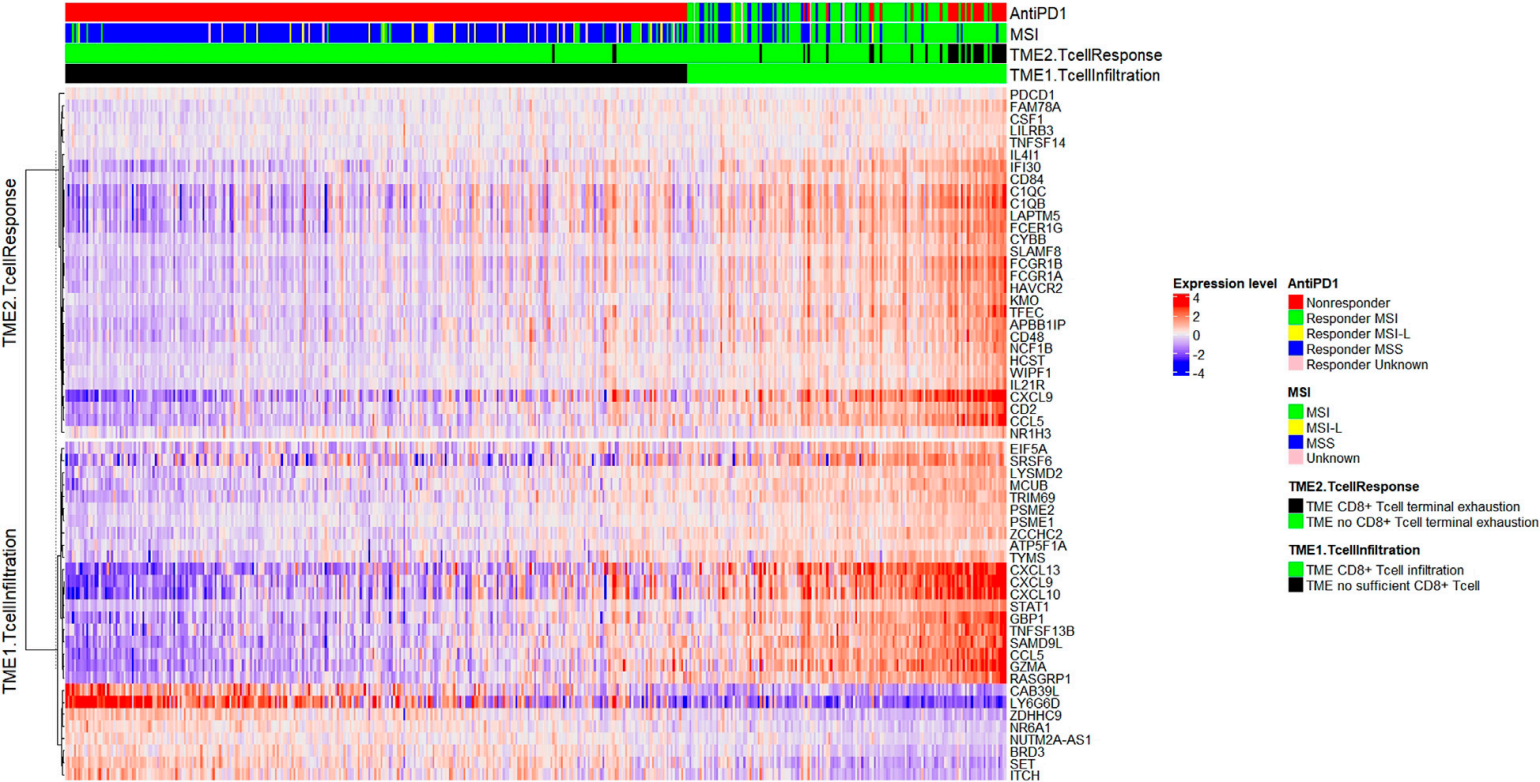
Plot of TMEPRE over 454 colorectal cancer samples (MSI = 131, MSI-L = 23, MSS = 284, Unknown = 16). Samples were ranked according to scores of the TMEPRE model. Genes in TME1.TcellInfiltration were shown in the upper panel and genes in TME2.TcellResponse were shown in the lower panel. MSI row indicates MSI/MSS status. TME1.TcellInfiltration row indicates TME1.TcellInfiltration scores, green indicates sufficient CD8^+^ T-cell infiltration, and black indicates insufficient CD8^+^ T-cell infiltration. TME2.TcellResponse row indicates TME2.TcellResponse scores: green indicates that infiltrated CD8^+^ T cells are not terminally exhausted if a tumor sample has CD8^+^ T cells infiltration and black indicates infiltrated CD8^+^ T cells are terminally exhausted if a tumor sample has CD8^+^ T cells infiltration. AntiPD1 indicates whether the tumor microenvironment of a sample displays biological characteristics that can respond to anti-PD1 treatment (red: nonresponder; green: MSI responder; blue: MSS responder). 10.6% of MSS patients and 67.2% of MSI patients could potentially benefit from anti-PD1 treatment. Within MSI nonresponders, approximately 50% showed insufficient amount of tumor-infiltrating CD8^+^ T cells and 50% showed terminal exhaustion of CD8^+^ T cells.

The TMEPRE model was read out in 454 colorectal samples (MSI = 131, MSI-L = 23, MSS = 284, Unknown = 16). A splitted heatmap was plotted (Gu et al., 2016). Tumors displaying a pattern of sufficient CD8^+^ T-cell infiltration but no pattern of CD8^+^ T-cell terminal exhaustion are considered as potential responders to anti-PD1 therapy (Figure 4).

Within 284 MSS tumor samples, 10.6% (n = 30) are classified as responders and 89.4% (n = 254) as nonresponders. This predicted percentage of responders, 10.6% by TMEPRE, is consistent with the reported disease control rate, 11%, in pembrolizumab-treated metastatic MSS colorectal cancers (Le et al., 2015). Among MSS nonresponders, 86.6% (n = 246) showed insufficient tumor-infiltrating CD8^+^ T cells and 2.8% (n = 8) showed sufficient tumor-infiltrating CD8^+^ T cells; however, those CD8^+^ T cells display patterns of terminally exhausted CD8^+^ T cells. As expected, the anti-PD1 resistance mechanism of the majority of MSS tumors is an insufficient amount of tumor-infiltrating CD8^+^ T cells.

Within 131 MSI tumor samples, 67.2% (n = 88) are classified as responders and 32.8% (n = 43) as nonresponders. This predicted percentage of responders, 67.2% by TMEPRE, is between the range of the reported immune-related objective response rate, 40%, and the reported disease control rate, 78%, in pembrolizumab-treated metastatic MSI colorectal cancers (Le et al., 2015). Among the MSI nonresponders, 16.0% (n = 21) showed insufficient tumor-infiltrating CD8^+^ T cells and 16.8% (n = 22) showed sufficient tumor-infiltrating CD8^+^ T cells; however, those CD8^+^ T cells display patterns of terminally exhausted CD8^+^ T cells. Therefore, approximately 50% of MSI nonresponders are caused by terminal exhaustion of CD8^+^ T cells in the tumor microenvironment, and the rest 50% of MSI nonresponders are caused by an insufficient amount of tumor-infiltrating CD8^+^ T cells. Treatments of MSI nonresponders with insufficient infiltration of CD8^+^ T cells and MSI nonresponders with terminal exhaustion of CD8^+^ T cells need to be designed separately.

The TMEPRE model identified 10.6% of MSS and 67.2% of MSI colorectal cancer patients whose tumors show biological characteristics that can potentially benefit from anti-PD1 treatment. These predicted percentages of responders in MSS tumors and MSI tumors are consistent with the reported benefits of immune-related disease control rate at 20 weeks of a cohort of colorectal cancer patients treated with pembrolizumab (Le et al., 2015).

## Discussion

Drug resistance tumors in colorectal cancer often consist of heterogeneous subgroups of populations. A drug response prediction method needs first to identify different drug resistance patterns of subgroups and then reinforce the patterns later (Tian et al., 2013; Tian et al., 2020). Conceptually, a drug response prediction method must never consider the drug resistance tumors as a homogenous group. Current biomarkers for anti-PD1 in colorectal cancer, MSI/MSS, TMB, PDL1, and POLE/POLD1 mutation, share the same notion that anti-PD1 resistance is dominantly caused by one homogenous factor of an insufficient amount of CD8^+^ T-cell infiltration. The quantity of tumor-infiltrating CD8^+^ T cells needs to be supplemented by characteristics of tumor-infiltrating CD8^+^ T cells to predict anti-PD1 response. In this report, we developed the computational method TMEPRE for colorectal cancer patients, which measures two factors of the tumor microenvironment that contribute to anti-PD1 resistance: CD8^+^ T-cell infiltration (TME1.TcellInfiltration) and whether tumor-infiltrating CD8^+^ T cells can respond to cancer immunotherapy (TME2.TcellResponse). TMEPRE was developed without using any anti-PD1 response data or survival data and was designed to reflect the biology of the tumor microenvironment of colorectal cancer. The method was validated in three datasets of anti-PD1-treated patients.

Another example of a prediction method of anti-PD1 response using tumor microenvironment of CD8^+^ T-cell exclusion and CD8^+^ T-cell exhaustion is TIDE (Jiang et al., 2018). The TIDE method also has good validation performance in anti-PD1-treated melanoma data. It is difficult to directly compare TIDE and TMEPRE as these two methods are optimized for different tumor types. TIDE method was trained using survival data of melanoma and was specifically designed for five cancer types: melanoma, neuroblastoma, triple-negative breast cancer, endometrial cancer, and acute myeloid leukemia. On the contrary, the TMEPRE method is designed for colorectal cancer. Partially because the methods are optimized for different cancer types, the overlap between genes used in the TMEPRE model and genes used in the TIDE model is small (IL21R, GZMA). We expect that TMEPRE will be a more specific reflection of the tumor microenvironment of colorectal cancer.

The first component of TMEPRE, TME1.TcellInfiltration, measures the tumor microenvironment that allows CD8^+^ T-cell infiltration. Approximately 50% of the MSI nonresponders have a low TME1.TcellInfiltration score. For those colorectal cancer patients, a combination of anti-PD1 with drugs inducing CD8^+^ T-cell infiltration could be considered (Duan et al., 2020). The second component of TMEPRE, TME2.TcellResponse, measures the tumor microenvironment and whether tumor-infiltrating CD8^+^ T cells can still respond to checkpoint inhibitors. It should be noted that because gene expression data were generated using bulk tumor samples, genes listed in TME2.TcellResponse are expressed on both CD8^+^ T cells and other immune cell types in the tumor microenvironment. Among the genes in TME2.TcellResponse, two genes are known inhibitor receptors on CD8^+^ T cells (HAVCR2, PDCD1) and seven genes (CCL5, CD2, CD48, CD84, FAM78A, HCST, and IL21R) have high expression levels in purified CD8^+^ T cells. The expression values of these nine genes are higher in nonresponders, which correlates with the terminally exhausted type of CD8^+^ T cells. Among the other genes in TME2.TcellResponse, CIQB, CIQC, KMO, FCGR1A, FCGR1B, and FCER1G show higher expression in nonresponders, and these are potential markers of the existence of myeloid-derived suppressor cells (Giorgini et al., 2013; Bournazos et al., 2016; Ryan et al., 2020). This pattern suggests that the reason why terminally exhausted CD8^+^ T cells failed to respond to anti-PD1 therapy might be not only the co-expression of multiple inhibitors on CD8^+^ T cells themselves but also the tumor microenvironment of colorectal tumor in which those terminally exhausted CD8^+^ T cells may be infiltrated with myeloid-derived suppressor cells. Approximately 50% of the MSI nonresponders have a high TME1.TcellInfiltration score but a low TME2.TcellResponse score. For those colorectal cancer patients, a combination of anti-PD1 with drugs targeting myeloid-derived suppressor cells or a combination of drugs targeting other co-expressed inhibitors could be considered (Kuang et al., 2020; Lind et al., 2020; Zhang et al., 2020).

By only assessing the MSI/MSS status, it is not recommended that MSS colorectal cancer patients be treated with anti-PD1. However, data from clinical trials showed that the disease control rate in pembrolizumab-treated metastatic MSS colorectal cancers was 11%, and further, in a more recent clinical trial of neoadjuvant setting, the pathological response rate of ipilimumab + nivolumab–treated early-stage MSS colorectal cancers is 27% (Le et al., 2015; Chalabi et al., 2020). These results indicated that responders to anti-PD1 treatment exist within the MSS colorectal cancer population. In our analysis, approximately 10.6% of MSS tumor samples showed both high TME1.TcellInfiltration scores and high TME2.TcellResponse scores, suggesting the biological characteristics of tumor microenvironments in 10.6% of MSS patients can still potentially benefit from anti-PD1 treatment. This prediction agrees with the reported immune-related progression survival rate of MSS patients treated with pembrolizumab. As the number of MSS patients is much larger than MSI patients, in this dataset of 451 patients used for this study, 10.6% of MSS patients means that the percentage of patients who should be considered for anti-PD1 treatment would increase 23%. The limitation is that, at this moment, no dataset of colorectal cancer patients treated with anti-PD1 is publicly available. A clinical trial is proposed at our cancer center to further confirm the prediction of TMEPRE in colorectal cancer.

To conclude, we develop a colorectal cancer-specific method, TMEPRE, that predicts cancer immunotherapy response. The global patterns of TMEPRE in colorectal cancer patients explained the mechanism underlying the response of anti-PD1 in MSS patients and the resistance of anti-PD1 in MSI patients. A subset of MSS patients could potentially benefit from anti-PD1 treatment. Anti-PD1-resistant MSI patients could result from tumor microenvironment of insufficient infiltration of CD8^+^ T-cell or tumor microenvironment of terminal exhaustion of CD8^+^ T cells, and treatment strategies need to be different. TMEPRE will aid personalized medicine options of cancer immunotherapy for colorectal cancer patients.

## Data Availability

The datasets presented in this study can be found in online repositories. The names of the repository/repositories and accession number(s) can be found in the article/Supplementary Material.
