# Supplementary material for: Global Pattern of CD8+ T-Cell Infiltration and Exhaustion in Colorectal Cancer Predicts Cancer Immunotherapy Response
Source: Front Pharmacol. 2021 Sep 10;12:715721. doi: 10.3389/fphar.2021.715721 (PMC8477790; doi:10.3389/fphar.2021.715721)
Supplement: Supplementary file 1 [file Table1.pdf]

Supplementary Table 1. List of genes in TME1.TcellInfiltration

Gene	GeneName	Occurrence CrossValidation	Nonresponder -Responder (log2)
ATP5F1A	ATP synthase F1 subunit alpha	185	-0.631853709932745
BRD3	bromodomain containing 3	200	0.894348826717143
CAB39L	calcium binding protein 39 like	194	1.62875575157413
CCL5	C-C motif chemokine ligand 5	200	-2.34053634275448
CXCL10	C-X-C motif chemokine ligand 10	200	-2.66553872474345
CXCL13	C-X-C motif chemokine ligand 13	200	-3.23749009278481
CXCL9	C-X-C motif chemokine ligand 9	200	-2.88457000691806
EIF5A	eukaryotic translation initiation factor 5A	198	-1.26804219626504
GBP1	guanylate binding protein 1	200	-2.05105194054728
GZMA	granzyme A	200	-2.54925523504469
ITCH	itchy E3 ubiquitin protein ligase	164	1.19046558320517
LY6G6D	lymphocyte antigen 6 family member G6D	200	2.74930943929956
LYSMD2	LysM domain containing 2	200	-1.06873387968098
MCUB	mitochondrial calcium uniporter dominant negative beta subunit	200	-1.1317217043933
NR6A1	nuclear receptor subfamily 6 group A member 1	183	0.719132295925746
NUTM2A-AS1	NUTM2A antisense RNA 1	200	0.598863541528726
PSME1	proteasome activator subunit 1	166	-0.726776305596763
PSME2	proteasome activator subunit 2	200	-0.865707282447989
RASGRP1	RAS guanyl releasing protein 1	200	-1.85917133104565
SAMD9L	sterile alpha motif domain containing 9 like	186	-1.93061174348838
SET	SET nuclear proto-oncogene	200	1.51366747633733
SRSF6	serine and arginine rich splicing factor 6	200	-1.96448919455866
STAT1	signal transducer and activator of transcription 1	200	-1.29882655260805
TNFSF13B	TNF superfamily member 13b	198	-1.87380016762539

TRIM69	tripartite motif containing 69	200	-1.13826341227555
TYMS	thymidylate synthetase	200	-1.12990316021394
ZCCHC2	zinc finger CCHC-type containing 2	200	-1.02605019051503
ZDHHC9	zinc finger DHHC-type containing 9	200	1.12912590423752
