# Supplementary material for: Global Pattern of CD8+ T-Cell Infiltration and Exhaustion in Colorectal Cancer Predicts Cancer Immunotherapy Response
Source: Front Pharmacol. 2021 Sep 10;12:715721. doi: 10.3389/fphar.2021.715721 (PMC8477790; doi:10.3389/fphar.2021.715721)
Supplement: Supplementary file 2 [file Table2.pdf]

Supplementary Table 2. List of genes in TME2.TcellResponse

Gene	GeneName	Occurrence CrossValidation	Nonresponder - Responder (log2)	CD8+Tcell
APBB1IP	amyloid beta precursor protein binding family B member 1 interacting protein	200	0.956828729546694	0
C1QB	complement C1q B chain	195	1.26537310535927	0
C1QC	complement C1q C chain	178	1.34214786223453	0
CCL5	C-C motif chemokine ligand 5	199	1.43929321169031	1
CD2	CD2 molecule	200	1.18579467326728	1
CD48	CD48 molecule	176	0.946309313953731	1
CD84	CD84 molecule	200	0.834765378113796	1
CSF1	colony stimulating factor 1	171	0.400737770390341	0
CXCL9	C-X-C motif chemokine ligand 9	193	1.65350805292663	0
CYBB	cytochrome b-245 beta chain	197	0.890276555946401	0
FAM78A	family with sequence similarity 78 member A	200	0.717743601056077	1
FCER1G	Fc fragment of IgE receptor Ig	194	1.00358292233769	0
FCGR1A	Fc fragment of IgG receptor Ia	200	1.18131963670012	0
FCGR1B	Fc fragment of IgG receptor Ib	200	1.31091446499147	0

HAVCR2	hepatitis A virus cellular receptor 2	200	1.02250648320338	1
HCST	hematopoietic cell signal transducer	200	0.710038811216336	1
IFI30	IFI30 lysosomal thiol reductase	199	0.81554481463394	0
IL21R	interleukin 21 receptor	200	0.924425668165048	1
IL4I1	interleukin 4 induced 1	198	0.69884237218103	0
KMO	kynurenine 3-monooxygenase	197	0.970911272597387	0
LAPTM5	lysosomal protein transmembrane 5	200	0.913042603326339	0
LILRB3	leukocyte immunoglobulin like receptor B3	179	0.434520931712665	0
NCF1B	neutrophil cytosolic factor 1B pseudogene	182	0.887589896558749	0
NR1H3	nuclear receptor subfamily 1 group H member 3	200	0.877093303833577	0
PDCD1	programmed cell death 1	200	0.426630128549923	1
SLAMF8	SLAM family member 8	199	0.725610426305764	0
TFEC	transcription factor EC	166	1.19651432817546	0
TNFSF14	TNF superfamily member 14	187	0.590408507927128	0
WIPF1	WAS/WASL interacting protein family member 1	199	0.736817599073468	0
